# Bone mineral density, body mass index and cigarette smoking among Iranian women: implications for prevention

**DOI:** 10.1186/1471-2474-6-34

**Published:** 2005-06-24

**Authors:** Azam Baheiraei, Nicholas A Pocock, John A Eisman, Nguyen D Nguyen, Tuan V Nguyen

**Affiliations:** 1 Bone and Mineral Research Program, Garvan Institute of Medical Research, St Vincent's Hospital, University of New South Wales, Sydney, Australia; 2 Department of Nuclear Medicine, St Vincent's Hospital, Sydney, Australia

## Abstract

**Background:**

While risk factors of osteoporosis in Western populations have been extensively documented, such a profile has not been well studied in Caucasians of non-European origin. This study was designed to estimate the modifiable distribution and determinants of bone mineral density (BMD) among Iranian women in Australia.

**Methods:**

Ninety women aged 35 years and older completed a questionnaire on socio-demographic and lifestyle factors. BMD was measured at the lumbar spine (LS) and femoral neck (FN) using DXA (GE Lunar, WI, USA), and was expressed in g/cm^2 ^as well as T-score.

**Results:**

In multiple regression analysis, advancing age, lower body mass index (BMI), and smoking were independently associated with LS and FN BMD, with the 3 factors collectively accounting for 30% and 38% variance of LS and FN BMD, respectively. LS and FN BMD in smokers was 8% lower than that in non-smokers. Further analysis of interaction between BMI and smoking revealed that the effect of smoking was only observed in the obese group (*p *= 0.029 for LSBMD and *p *= 0.007 for FNBMD), but not in the overweight and normal groups. Using T-scores from two bone sites the prevalence of osteoporosis (T-scores ≤ -2.5) was 3.8% and 26.3% in pre-and post-menopausal women, respectively. Among current smokers, the prevalence was higher (31.3%) than that among ex-smokers (28.6%) and non-smokers (7.5%).

**Conclusion:**

These data, for the first time, indicate that apart from advancing age and lower body mass index, cigarette smoking is an important modifiable determinant of bone mineral density in these Caucasians of non-European origin.

## Background

Osteoporosis is a common disorder in the elderly population, and represents one of the most significant public health problems in the world, predisposing to fractures with minimal or no antecedent trauma. These fractures are, in turn, associated with increased morbidity [1], reduced quality of life [2], mortality [3], and high health care costs [4].

Bone mineral density (BMD) measurement is considered an effective predictor of fracture risk, such that each standard deviation lower in BMD is associated with at least a 2-fold increase in age adjusted fracture risk. [5-7]. Therefore, a useful approach in assessing the importance of aetiological factors for osteoporosis is an investigation of the distribution and determinants of BMD. Although determinants of bone mineral density in Western populations have been extensively studied, such a profile has not been well documented in Caucasians of non-European origin.

Body weight or body mass index (BMI) is known to be positively associated with BMD[8,9]. Lifestyle factors such as low calcium intake, lack of physical activity, and smoking adversely affect bone mineral density and increase the risk of osteoporosis and its related fractures[10]. These factors also play an important role in the determination of peak bone mass and subsequent bone loss during the post-menopausal period. Among the modifiable risk factors of osteoporosis, cigarette smoking is considered one of the deleterious factors because cigarette smokers also have increased risk of fracture[11,12]. Nevertheless, the interactive effect of smoking on BMD has not been well studied. A recent study in a Caucasian population suggested that the effect of smoking was modified by body mass index, such that non-obese smokers had lower BMD than obese-smokers[13]. Iranian women on the average have a relatively high BMI[14,15], and it is not known whether such an interaction effect between smoking and BMI is present in this population.

The present study was designed to examine the modifiable distribution and determinants of bone mineral density among Iranian Australian women.

## Methods

### Subjects and setting

This study was designed as a cross-sectional investigation. All women were recruited via a media campaign using newsletters, noticeboards in community halls as well as word of mouth at community centres as part of a larger study to examine osteoporosis prevention in Iranian women. Inclusion criteria for the study were Iranian women and aged 35 years or older. The exclusion criteria were: current or past occurrence of any medical conditions known to affect bone metabolism such as Paget's disease and stroke; current pregnancy; and/or a history of breastfeeding within the last year. Also excluded were women who had been taking any medication affecting bone such as hormones, calcium, and glucocorticoids. In total, 96 women participated in the current study. Six women, who did not meet study's criteria on the basis of diseases or history of taking medications affecting bone, were excluded from the analysis. This study was approved by the University of New South Wales's Human Research Ethics Committee and written informed consent was obtained from each participant.

### Data collection and measurements

#### Socio-demographic characteristics and lifestyle risk factors

Each woman completed a modified structured questionnaire [16] on socio-demographic and lifestyle risk factors. Income was included to be assessed, however, most participants refused to obtain information about their income level. Reproductive factors such as menopausal status and years since menopause were also provided for each participant. Menopause was defined as previous natural or surgical cessation of menstruation for more than 12 months. Calcium intake was calculated as the sum of current intake of main dairy products (milk, yogurt, and cheese) and was then converted to milligrams of calcium per day. Calcium contents for dairy products were provided from the product information in Australia [17]. Exercise was dichotomized as "yes" for current regular exercising, or "no" for not exercising. Amongst those who exercised, total amount of time spent per week was recorded. Current alcohol use was recorded as "yes" for drinking alcohol (beer, wine and liquor), or "no" for no intake of alcohol. Smoking habits were assessed based on previous and current cigarette smoking. Smoking status was dichotomized as "yes" for smoking, or "no" for never smoking. In addition, amongst those who smoked, dose and duration of smoking was recorded.

#### Anthropometric data

Weight (kg) and height (cm) were measured with light indoor clothing without shoes at the time of bone densitometry measurements. Weight was recorded to the nearest tenth of a kg using an electronic scale and standing height was measured to the nearest centimeter with a stadiometer. Body mass index (BMI) was calculated as body weight in kilograms divided by height in meters squared. According to the World Health Organization (WHO) recommended classification system, overweight and obese individuals were classified as having a BMI between 25 and 29, and equal to or greater than 30 kg/m^2^, respectively[18].

#### Bone density measurement

BMD was measured at the lumbar spine (LS) (L2-L4, anterior-posterior position) and femoral neck (FN) using dual-energy X-ray absorptiometry (DXA) with a Lunar Prodigy densitometer (GE Lunar, WI, U.S.A.). Areal BMD was expressed in g/cm^2 ^and in standard deviations from the young normal mean (T-score), based on the Australian Reference Population. The sample of women was grouped into 3 groups based on the WHO recommended criteria: osteoporosis if T-score ≤ -2.5; osteopenia if -2.5 < T-score ≤-1.0; and normal if T-score >-1.0 [19].

### Data analysis

To determine the magnitude of association between the potential risk factors (e.g., menopausal status, height, weight, dairy calcium intake, smoking, exercise, and alcohol use) and osteoporosis risk. Bone mineral density was considered the primary outcome, and was treated as a continuous variable. Individual risk factors were first considered in a simple linear regression analysis to estimate the strength of association between individual risk factor and BMD. In the subsequent analysis, all risk factors were simultaneously considered in a multiple linear regression analysis using the backward elimination algorithms, to screen for independent significant factors. Residual analysis performed to ensure that the usual assumptions of the regression model (i.e. normality, homogeneity and independence) were met. The entry of significance level (*p *value) was set to 0.10 to arrive at the most robust model.

In further analysis, differences between the pre-menopausal and post-menopausal groups were tested by unpaired t-test for the normally distributed variables, or the Mann-Whitney U test for non-normally distributed variables, and Chi-square test for categorized data. The analysis was performed with the SAS statistical analysis system[20] and SPSS for Windows statistical software [21].

## Results

### Characteristics of study subjects

The study population consisted of 90 women aged 48.5 ± 8.3 yr (mean age ± SD; range: 35 to 77 yr). Approximately 42 % of the women had education within high school. The majority of the women were married (78%) and performing home duties or not employed (56%). Their average duration of residence in Australia was about 10 years, with 75% of subjects having resided in Australia for at least 5 years. The mean age (SD) at immigration was about 39 ± 9.4 yr (range: 18 to 65 yr). The median (SD) dairy calcium intake in the women was 407 ± 283 mg/day. The Twenty-three women (26%) exercised regularly. Approximately 26% of women smoked cigarettes during their lifetime. Although cigarette smoking was common in these subjects, alcohol use was not frequent with about 11% of the women reporting drinking any kind of alcohol. Using the BMI criteria, 2.2% of subjects were underweight; 25.6% of women were in the healthy weight range; 35.6% were over-weight; and 36.7% were obese.

Forty two percent (n = 38) of women were post-menopausal, with the duration of post-menopause being between 1 and 32 years. Post-menopausal women had significantly higher age and parity and lower height, lumbar spine and femoral neck BMD, but no significant differences were found between the pre-and post-menopausal women in weight, BMI, dairy calcium intake, exercise, smoking status, duration of smoking, and alcohol use (Table 1).

**Table 1:** Clinico-demographic characteristic of study subjects

	**Pre-menopause**	**Post-menopause**	**p value**
N	52	38	
Age (years)*	43.6 ± 4.7	55.18 ± 7.4	<0.001^a^
Height (cm)*	157.7 ± 5.5	155 ± 5.7	0.027^a^
Weight (kg)*	70.8 ± 16.4	68.9 ± 10.3	0.530^a^
BMI (kg/m^2^)*	28.5 ± 6.9	28.7 ± 4.3	0.869^a^
LSBMD (g/cm^2^)*	1.19 ± 0.15	1.04 ± 0.16	<0.001^a^
FNBMD (g/cm^2^)*	0.97 ± 0.12	0.87 ± 0.11	<0.001^a^
Dairy calcium intake (mg/day)*	410 ± 262	498 ± 306	0.147^a^
Age at menopause*	-	47.9 ± 4.02	-
Parity^†^	2 (2, 3)	3 (2, 4)	0.004^b^
Regular exercise^§^	12 (23.1)	11(28.9)	0.528^c^
Smoking status			
Current smokers	15.4 (8)	21.1 (8)	0.487^c^
Ex-smokers	21.2 (11)	23.7 (9)	0.775^c^
Duration of smoking (years)^§^			0.155^c^
≤ 5	4 (33.3)	1 (9.1)	
> 5	8 (66.7)	10 (90.9)	
Alcohol use^§^	7 (13.5)	3 (7.9)	0.407^c^

*Mean ± SD; ^†^median (interquartile range); ^§ ^n (%).

^a^unpaired t-test, ^b^Mann-Whitney U test, ^c^Chi-square test.

BMI, body mass index; LSBMD, lumbar spine bone mineral density; FNBMD, femoral neck bone mineral density.

### Determinants of BMD

In simple linear regression analysis, age, height, weight, BMI, menopausal status, smoking habits, duration of smoking, and cigarette dose were each significantly associated with LS and FN BMD (Table 2). However, in the multiple linear regression, advancing age, lower BMI and smoking were independent predictors of LS and FN BMD (Table 3). After adjusting for age and BMI, smokers had 0.087 g/cm^2 ^(8 %) and 0.075 g/cm^2 ^(8 %) lower in LS and FN BMD, respectively, than non-smokers. The 3 factors collectively accounted for 30% and 38% of the variation in LS and FN BMD, respectively.

**Table 2:** Univariate association between individual risk factors and bone mineral density

**Factor**	**LSBMD (g/cm^2^)**		**FNBMD (g/cm^2^)**	
	β ± SE ^a^	R^2b^	β ± SE^a^	R^2b^
Age (per 1 year)	-0.009 ± 0.002*	0.20	-0.008 ± 0.001*	0.25
Height (per -5 cm)	0.04 ± 0.012*	0.07	0.030 ± 0.010*	0.07
Weight (per -5 kg)	0.02 ± 0.005*	0.10	0.015 ± 0.005*	0.14
BMI (per -5 kg/m^2^)	0.035 ± 0.015*	0.05	0.030 ± 0.010*	0.07
Post-menopause	-0.153 ± 0.033*	0.19	-0.103 ± 0.026*	0.15
Smoking (current and ex-smokers)	-0.100 ± 0.040*	0.06	-0.086 ± 0.030*	0.08
Duration of smoking (per 5 years)	-0.037 ± 0.015*	0.06	-0.031 ± 0.011*	0.07
Cigarette dose (per 10 cig/day)	-0.054 ± 0.019*	0.08	-0.038 ± 0.014*	0.07
Dairy calcium intake (per 300 mg/day)	0.020 ± 0.03	0.001	0.020 ± 0.03	0.02
Regular exercise (yes)	0.017 ± 0.042	0.002	0.044 ± 0.031	0.02
Alcohol use (yes)	0.004 ± 0.058	0.001	-0.021 ± 0.044	0.003

BMI, body mass index; LSBMD, lumbar spine bone mineral density; FNBMD, femoral neck bone mineral density.

* P < 0.05 (statistically significant)

^a ^Values are regression coefficients ± SE describing the change in bone mineral density (g/cm^2^) associated with the unit change in the risk factor

^b ^Coefficient of determination: the proportion of variation in bone mineral density explained by the variation in a risk factor

**Table 3:** Association between age, body mass index, smoking, and bone mineral density:Results of multiple linear regression analysis

**Determinant**	**LSBMD (g/cm^2^)^a^**	**FNBMD (g/cm^2^)^a^**
Age (per 1 year)	-0.008 ± 0.002**	-0.007 ± 0.001**
BMI (-5 kg/m^2^)	0.006 ± 0.003*	0.005 ± 0.002**
Smoking (yes)	-0.087 ± 0.035*	-0.075 ± 0.025**
R^2^*	0.30	0.38

*0.01 <*p *< 0.05; **0.0001 <*p *< 0.01

^a ^Values are regression coefficients ± SE describing the change in BMD (g/cm^2^) associated with one year advancing age, 5 kg/ m^2 ^decrease in BMI and smoking status.

BMI, body mass index; LSBMD, lumbar spine bone mineral density; FNBMD, femoral neck bone mineral density.

* R^2^, coefficient of determination: a measure of the proportion of variation in BMD explained by the variation in the risk factors. The variables included in the initial regression analysis were: age, menopause status, BMI, smoking status, duration of smoking, cigarette dose, calcium intake, exercise, and alcohol use

**Figure 1:**
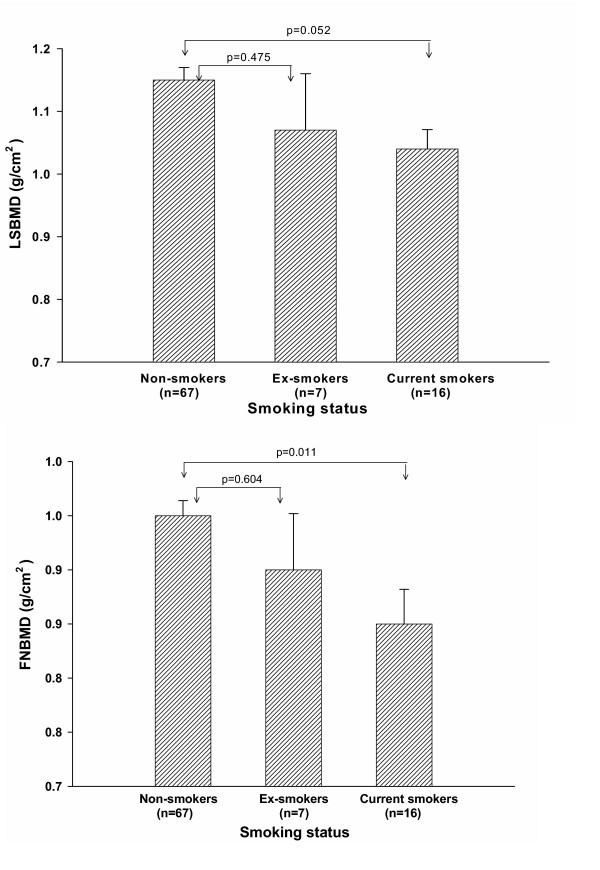
Mean and standard error of lumbar spine (upper panel) and femoral neck (lower panel) bone mineral density (g/cm^2^) by smoking status.

As expected, advancing age was negatively associated with BMD in the both sites (LS: *r *= 0.45, *p *= 0.0001; FN: *r *= 0.50, *p *= 0.0001). Nevertheless, there was a significant positive correlation between BMI and LS and FN BMD (LS: *r *= 0.22, *p *= 0.033; FN: *r *= 0.26, *p *= 0.012). Current smokers had significantly lower lumbar spine and femoral neck BMD than non-smokers. However, there was no significant difference between ex-smokers and non-smokers in both BMD sites (Fig. 1). Among smokers, there was no significant linear correlation between cigarette dose and BMD (*p *= 0.14 for LSBMD and *p *= 0.64 for FNBMD) and duration of smoking and BMD (*p *= 0.76 for LSBMD and *p *= 0.86 for FNBMD).

**Figure 2:**
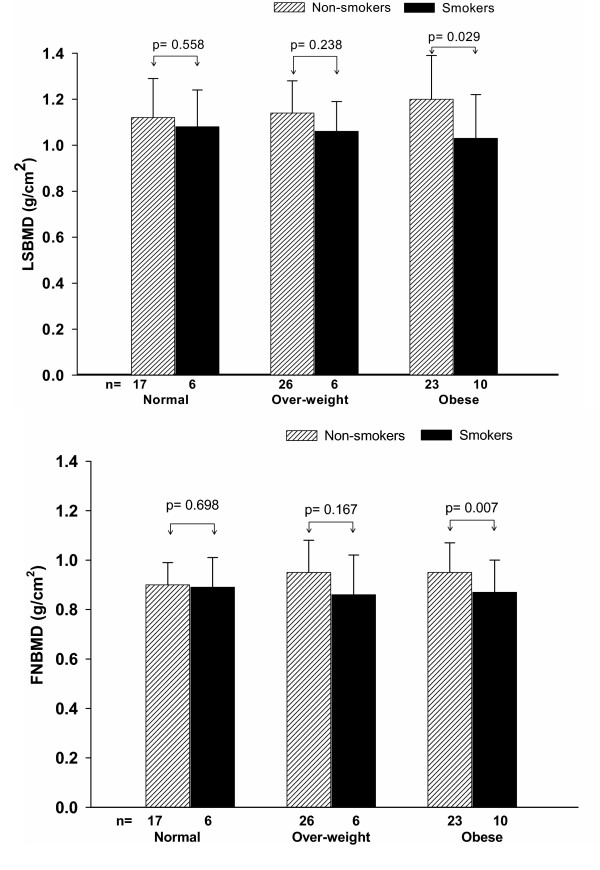
Mean and standard error of lumbar spine (upper panel) and femoral neck (lower panel) bone mineral density (g/cm^2^) by body mass index and smoking status.

**Figure 3:**
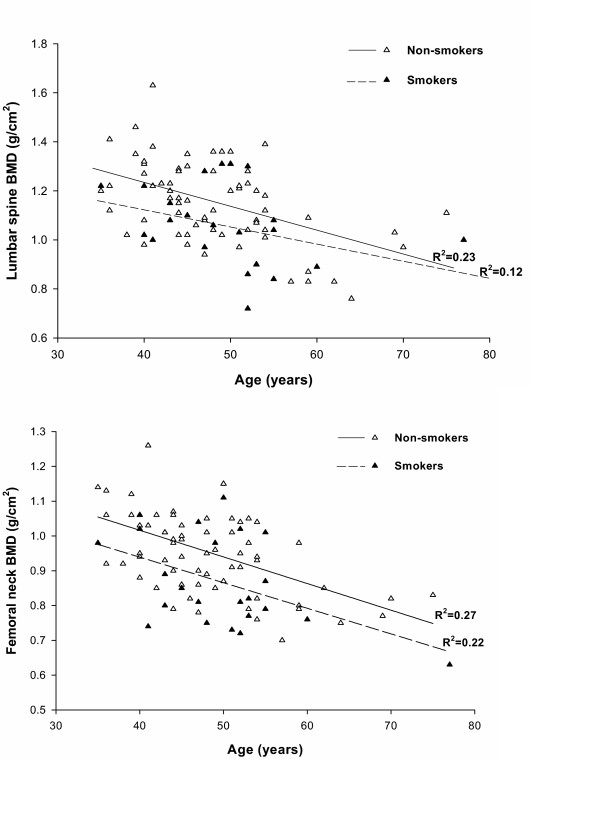
Interaction between age and smoking status on lumbar spine (upper panel) and femoral neck (lower panel) bone mineral density (g/cm^2^).

Further analysis of interaction between BMI and smoking revealed that the effect of smoking was only observed in the obese group (*p *= 0.029 for LSBMD and *p *= 0.007 for FNBMD), but not in the overweight and normal groups (Fig. 2). This interaction effect was not affected by the dose of cigarette or duration of smoking. Moreover, there was a non-statistically significant interaction between age and smoking, as both smokers and non-smokers appeared to have a similar age-BMD association (Fig. 3).

### Prevalence of low bone density

Twenty-five women (27.8%) were osteopenic (T-score -1 to -2.49) at the lumbar spine and 32 (35.6%) at the femoral neck. Using the WHO T-score-based definition of osteoporosis, the proportion of women with osteoporosis was 12.2% (n = 11) at the lumbar spine and 2.2% (n = 2) at the femoral neck. When the two measures were considered simultaneously, the prevalence of osteoporosis was 13.3%. In post-menopausal women, the prevalence of osteoporosis (T-score ≤ -2.5) was 23.7% (n = 9) at the lumbar spine and 5.3% (n = 2) at the femoral neck. Among smokers, the prevalence was 30.4% (7/23) which was significantly higher (*p *< 0.01) than that among non-smokers (7.5%, 5/67).

## Discussion

Osteoporosis is recognized as a public health problem in the world, but its epidemiology in non-Western populations remains poorly understood. This study represents an original contribution to the study of osteoporosis in Iranian women in Australia. Apart from advancing age and body mass index, it was found that cigarette smoking was an important modifiable determinant of bone mineral density.

These results are consistent with previous studies which indicated that advancing age was associated with lower BMD. In this study, each year increase in age was estimated to cross-sectionally "decrease" 0.8% in LS and FN BMD. This estimate is relatively consistent with longitudinal studies in Western Caucasian women which suggest an annual decrease of 1%[22,23].

Consistent with previous studies[8,9], this study found that lower BMI was associated with lower BMD. Moreover, the effect of smoking was significant in obese women. The prevalence of current cigarette smoking in this population (17.8%) was surprisingly higher than Iranian women in Iran which was around 3.6%[24]. BMD in smokers was 8% lower than in non-smokers, after adjusting for age and BMI. This difference is clinically significant, because each SD reduction in BMD is associated with a 2-fold increase in age adjusted fracture risk.

BMD measurements in ex-smokers were intermediate between current and non-smokers. It seems the effect of smoking varies linearly with the intake of cigarette and may suggest that smoking cessation have a positive effect on both LS and FN BMD. This makes cigarette smoking one of the important modifiable lifestyle risk factors of osteoporosis in Iranian Australian women.

Although some studies found an association between calcium intake and BMD, the present study found no such association. The results also revealed a non-significant relationship between exercise and BMD. One of the reasons for these results may be that the questionnaire only reflected the present situation, not permanent lifestyle. In addition, frequency and type of exercise on bone density could not be evaluated, because few of the subjects exercised regularly.

Alcohol use was not significantly associated with bone mineral density. Although cigarette smoking was common in this sample, alcohol use was not frequent and only a few (11%) women reported drinking any kind of alcohol, with the majority drinking only monthly or rarely. With such a low prevalence of use, it is perhaps not surprising that the study was unable to detect a significant effect of alcohol use on BMD.

Using T-scores from two bone sites the prevalence of osteoporosis (T-scores ≤ -2.5) was 3.8% (n = 2) and 26.3% (n = 10) in pre-and post-menopausal women, respectively. The most notable observation in this study is that osteoporosis was more likely to be identified at the lumbar spine than the femoral neck. This finding is consistent with a previous study among Iranian women in Iran [25]. However, the prevalence of osteoporosis at the femoral neck seemed to be relatively lower in the Iranian women compared with most Asian and other Caucasian populations [26-29]. These results suggest that BMD measured at the femoral neck requires further investigation in Iranian women.

The present study's findings must be interpreted within the context of a number of strengths and weaknesses. This study is the first attempt to address an important public health problem amongst Iranian women in an Australian setting. Some aspects of acculturation should be taken into account in the interpretation of these findings. Since 75% of women have settled in Australia for at least 5 years, change of lifestyle factors such as sun exposure and diet cannot be ruled out, and the present results may not be generalizable to Iranian women in Iran. The association between risk factors and BMD as observed in this study cannot be interpreted as a causal relationship because the study was a cross-sectional investigation. Because the women in this study were not sampled from an age-stratified scheme, the average T-scores and prevalence of osteoporosis could have been affected by the actual age group distribution, and this represents a potential limitation for generalizing the results to the general population.

## Conclusion

These data suggest that, apart from advancing age and lower BMI, cigarette smoking is an important modifiable determinant of bone mineral density in the Iranian Australian women. These findings can potentially contribute toward the development of more effective public health strategies for the health promotion and osteoporosis prevention in this population.

Further studies are required to investigate the effect of changing environmental exposures which can influence osteoporosis prevalence and fracture risk in this population.

## Competing interests

The author(s) declare that they have no competing interests.

## Authors' contributions

Conception and study design: AB, JE, TN

Data collection: AB, NP

Drafting manuscript: AB

Data analysis: AB, NN, TN

Review of manuscript for important intellectual content: AB, NP, JE, TN

All authors read and approved the final manuscript.

## Pre-publication history

The pre-publication history for this paper can be accessed here:


